# A single center experience of concomitant administration of sofosbuvir/velpatasvir and amiodarone after heart and lung transplantation

**DOI:** 10.1016/j.jhlto.2025.100279

**Published:** 2025-05-02

**Authors:** Ryan M. Rivosecchi, Pablo G. Sanchez, Lauren M. Sacha, Edward T. Horn, Mary Keebler, Fernanda P. Silveira

**Affiliations:** a Department of Pharmacy, UPMC Presbyterian, 200 Lothrop St., Pittsburgh, PA 15213; b Department of Cardiothoracic Surgery, UPMC Presbyterian, 200 Lothrop St., Pittsburgh, PA 15213; c Department of Pharmacy and Therapeutics, University of Pittsburgh School of Pharmacy, 550 Scaife Hall, 3550 Terrace Street, Pittsburgh, PA 15213; d Department of Medicine, Division of Cardiology, University of Pittsburgh, 200 Lothrop St C-700, Pittsburgh, PA 15213; e Department of Medicine, Division of Infectious Diseases, University of Pittsburgh, 3601 Fifth Avenue Suite 5B, Pittsburgh, PA 15213

**Keywords:** Hepatitis C, Amiodarone, Bradycardia, Heart transplant, Lung transplant

## Abstract

The advent of direct-acting antiviral agents (DAA) allows for the utilization of hepatitis C (HCV) viremic donors to uninfected recipients. Sofosbuvir/velpatasvir (SOF/VEL) is a pangenotypic DAA without clinically significant interactions with immunosuppressants, but its concomitant use with amiodarone may cause serious bradycardia. In an open-label study of 20 heart and lung transplant recipients of organs from HCV viremic donors, who received SOF/VEL for 12 weeks starting on post-operative day one, the concomitant use of amiodarone and SOF/VEL resulted in bradycardic episodes and commonly occurred in the presence of beta-blocking medications. Episodes of bradycardia were transient, resolved with intervention, and did not require the discontinuation of SOF/VEL. In patients requiring the combination of SOF/VEL and amiodarone, consideration of therapeutic options is warranted prior to the addition of other bradycardia-inducing medications.

## Background

According to data from the Health Resources and Services Administration, more than 100,000 people are on the solid organ transplant waitlist in 2024.^1^ Despite advances in transplantation, the number of suitable donor organs remains a limiting factor, and waitlist mortality remains substantial.^1^ In the United States, there has been a dramatic increase in the number of organ donors who have died from drug intoxication, leading to an increase in transplantation.^2^ However, many medically suitable donor organs were left unused due to infection with hepatitis C virus (HCV).^2^, ^3^

The development of direct-acting antiviral agents (DAAs), including sofosbuvir/velpatasvir (SOF/VEL), has allowed transplant centers to utilize organs from HCV viremic donors into uninfected recipients. Several studies in abdominal and thoracic transplantation have shown this practice to be safe and effective,^4^, ^5^, ^6^, ^7^ and it has become standard of care at many transplant centers.

There are important drug-drug interactions in the post-transplant setting. The concomitant usage of amiodarone and sofosbuvir may cause serious symptomatic bradycardia and is not recommended by the manufacturer.^8^ Sofosbuvir inhibits P-glycoprotein, a transporter protein responsible for the elimination of amiodarone. This may increase the concentration of amiodarone, enhancing its bradycardic effects. Thoracic transplant recipients experience an elevated risk of atrial arrhythmias in the immediate post-transplant period, with nearly a third of lung transplant recipients and up to 40% of heart transplant recipients developing arrhythmia.^9^, ^10^ Many clinicians choose amiodarone for the management of arrhythmia in this period due to hemodynamic challenges that preclude the use of alternatives.

The aim of this study was to report our experience with the concomitant administration of SOF/VEL and amiodarone in heart and lung transplant recipients.

## Materials and methods

This was a retrospective review of patients prospectively enrolled in an open-label study of transplantation of hearts and lungs from HCV viremic donors to HCV negative recipients at UPMC Presbyterian Hospital. HCV seronegative adults 18 years of age or older who received heart or lung transplantation from a HCV viremic donor were given SOF/VEL (400/100 mg) starting on post-operative day 1 and continued one time daily for 12 weeks. If the patient was unable to swallow SOF/VEL, it was crushed and administered via enteral feeding tube. Patients with seropositivity for HIV, HCV, or hepatitis B surface antigen; a history of cirrhosis or pre-existent liver disease; persistently elevated liver enzymes prior to transplant; presence of atrial tachyarrhythmias; or on extra-corporeal membrane oxygenation preceding transplant were excluded. Patients receiving at least one dose of amiodarone, intravenous or enteral, were included. The initiation and management of amiodarone were determined by the multidisciplinary treatment team, as were other cardiovascular medications.

The outcome of interest was the development of bradycardia, defined as a heart rate less than 60 beats per minute. Sustained bradycardia was defined as a heart rate <60 beats per minute for at least five consecutive minutes. Lastly, we considered any change in care to the patient relative to heart rate to be a bradycardic event requiring intervention.

Discrete variables were expressed as number (*n*) and percentages. Continuous variables were reported as median and interquartile ranges (IQR). The study was approved by the University of Pittsburgh Institutional Review Board.

## Results

Twenty thoracic transplant recipients (10 lung and 10 heart) from an HCV viremic donor received SOF/VEL in this open-label protocol. Among them, 11 (55%, eight lung and three heart) transplant recipients concomitantly received at least a single dose of amiodarone. These 11 patients are described here. Four patients (36%) had a prior history of arrhythmia, and six patients (55%) received a beta-blocker prior to transplantation. Additional details on cardiac history and home medications are summarized in Table 1. No patients received amiodarone prior to transplantation.

**Table 1:** Demographics and Baseline Medical Characteristics

Demographics	*N* = 11
Male Sex	6 (55%)
Age, median (range)	65 (54-72)
*Organ Transplanted*	
Lung Heart	8 (73%) 3 (27%)
*Notable Cardiovascular History*	
Hypertension Coronary artery disease Heart failure Atrial fibrillation	6 (55%) 3 (27%) 3 (27%) 4 (36%)
*Notable home medications*	
Beta-blocker Calcium channel blocker Digoxin	6 (55%) 2 (18%) 1 (10%)

Amiodarone was initiated at a median of 2.5 days (IQR: 1.6-5.6) after transplant, with the median duration of concomitant administration of SOF/VEL and amiodarone being 30 days (IQR: 4-41). The median cumulative amiodarone dose was 9,086 mg (IQR: 2,195-10,952), with total amiodarone exposure ranging from 150 to 14,801 mg during co-administration. All patients received at least one bolus of intravenous amiodarone, and 10 received a continuous infusion. Seven patients (64%) continued enteral amiodarone during hospital admission, and three patients (27%) were discharged on amiodarone. Eight patients (73%) received a beta-blocker while on concomitant SOF/VEL and amiodarone (Table 2). There was no co-administration of additional rate or rhythm control medications.

**Table 2:** Sofosbuvir/Velpatasvir and Amiodarone Medication Administration Details

Details	*N* = 11
Transplant to SOF/VEL, days (IQR)	1.4 (1.3-1.5)
Transplant to amiodarone, days (IQR)	2.5 (1.6-5.6)
SOF/VEL to amiodarone, day (IQR)	1.2 (0.6-4.7)
Duration of amiodarone exposure, days (IQR)	25 (1-37)
Cumulative amiodarone dose, mg (IQR)	9,086 (2,195-10,952)
Concomitant SOF/VEL and amiodarone, days (IQR)	30 (4-41)

A total of 7,573 heart rates were documented during the co-administration of amiodarone and SOF/VEL. Only 90 charted values (1.2%) were bradycardic and ranged from 0% to 3.9% of documented heart rates for individual patients. Seven patients (63%) experienced at least one documented bradycardic event, with six patients (86%) requiring intervention and four patients (57%) experiencing sustained bradycardia (Table 3). Six out of the seven patients (85%) with at least one bradycardic event received a beta-blocker concomitantly with amiodarone. There were no discontinuations of SOF/VEL, nor readmissions for bradycardia.

**Table 3:** Bradycardic Events Among 11 Patients on Concomitant SOF/VEL and Amiodarone

Organ	Concomitant Beta-Blocker	Other Bradycardia Risk Medications	Cumulative Amiodarone (mg)	Bradycardia	Intervention
Lung	Yes	Midodrine	14,801	Yes	Re-warming of patient after operating room
Lung	Yes	Midodrine	9,086	Yes	Amiodarone discontinuation
Lung	Yes	No	2,363	Yes	Norepinephrine infusion, discontinuation of metoprolol and amiodarone
Lung	Yes	Midodrine	11,964	Yes	Metoprolol discontinuation
Lung	Yes	No	9,472	Yes	Amiodarone discontinuation
Heart	No	No	219	Yes	Increase in pacer setting
Lung	Yes	No	9,939	Yes	None
Lung	Yes	No	12,120	No	N/A
Lung	Yes	No	2,027	No	N/A
Heart	No	No	2,723	No	N/A
Heart	No	No	150	No	N/A

SOF/VEL, sofosbuvir/velpatasvir.

## Discussion

The advent of DAAs has allowed the transplant community to treat and cure HCV acquired through organ transplantation, therefore expanding the donor pool. As the donor’s HCV genotype is not readily available at the time of transplant, SOF/VEL is an attractive regimen because of its pangenotypic spectrum. The occurrence of post-transplant arrhythmias, which are often managed with amiodarone, could limit the utilization of SOF/VEL in the immediate post-thoracic transplant setting.

In our small series of 20 thoracic transplant recipients treated with SOF/VEL, 55% of transplant recipients required administration of at least one dose of amiodarone. Among those who received amiodarone, 73% received a beta-blocker. It is therefore not surprising that the majority of patients experienced at least one bradycardic episode. Bradycardia most commonly was managed with the discontinuation of amiodarone or beta-blockade, while the discontinuation of SOF/VEL was not required.

In the series by Woolley et al there were no clinically significant bradyarrhythmic episodes associated with sofosbuvir among only three patients on SOF/VEL who received amiodarone for management of atrial fibrillation post-thoracic transplant.^6^ Only grade III adverse events were reported in this series, while our definition of bradycardia was more liberal, and it appears that there was no concomitant use of beta-blockers in this cohort. In addition, the duration of concomitant administration of SOF/VEL and amiodarone was not reported, while in our series, the median duration of co-administration was 30 days.

Glecaprevir/pibrentasvir is another pangenotypic regimen that can be used to treat HCV-uninfected recipients of organs from HCV-viremic donors. Although it lacks the drug-drug interaction with amiodarone, making it an attractive option in the management of thoracic organ transplant recipients, there are other important drug-drug interactions between Glecaprevir/pibrentasvir and medications commonly used after thoracic transplantation, such as tacrolimus and statins. Besides, the choice of DAA is dictated by the institution’s formulary and cost.

Studies in HCV uninfected kidney and thoracic transplant recipients of organs from HCV viremic donors have shown that short and ultra-short duration DAA is safe and effective^6^, ^11^, ^12^ and drug-drug interactions such as SOF/VEL and amiodarone further support the case for shorter treatment durations.

## Conclusion

The concomitant use of amiodarone and SOF/VEL resulted in bradycardic episodes, commonly occurring in the presence of beta-blocking medications. Episodes were transient, resolved with intervention, and did not require the discontinuation of SOF/VEL. In patients requiring the combination of SOF/VEL and amiodarone, consideration of therapeutic options is warranted prior to the addition of other bradycardia-inducing medications.

## Disclosure Statement

The authors declare that they have no known competing financial interests or personal relationships that could have appeared to influence the work reported in this paper. Gilead Sciences Inc. donated the treatment courses of Epclusa (sofosbuvir/velpatasvir) for this study. Gilead had no role in relation to the study design, collection, analysis and interpretation of data, writing of the report, and decision to submit the article for publication.
